# Secreted frizzled-related protein 5 (SFRP5) protects ATDC5 cells against LPS-induced inflammation and apoptosis via inhibiting Wnt5a/JNK pathway

**DOI:** 10.1186/s13018-021-02260-5

**Published:** 2021-02-11

**Authors:** Minghui Sun, Weijun Wang, Lingtian Min, Cheng Chen, Qing Li, Wenjie Weng

**Affiliations:** 1 Department of Joint Surgery, The Affiliated Drum Tower Hospital of Nanjing University Medical School, No. 321 Zhongshan Road, Nanjing, 210008 China; 2 Department of Clinical Medicine of Chinese and Western Medicine Integration, Nanjing University of Chinese Medicine, Nanjing, 210023 China

**Keywords:** C-Jun N-terminal kinase, Inflammation, Osteoarthritis, Secreted frizzled-related protein 5, Wnt5a

## Abstract

**Background:**

Secreted frizzled-related protein 5 (SFRP5) is an endogenous inhibitor of Wnt5a (wingless-type family member 5a), which has been implicated in anti-inflammatory response. In this study, we aimed to investigate whether SFRP5 could protect chondrocytes against LPS-induced inflammation and apoptosis.

**Methods:**

ATDC5 cells that overexpressed with SFRP5 or not were challenged with LPS to observe the effects of SFRP5 overexpression on LPS-triggered inflammation and apoptosis as well as Wnt5a/JNK activation. Wnt5a was elevated in ATDC5 cells in the presence of SFRP5 overexpression, to determine whether Wnt5a/JNK signaling was involved in the actions of SFRP5.

**Results:**

The mRNA and protein levels of SFRP5 was significantly reduced by LPS in a concentration-dependent manner. Overexpression of SFRP5 in ATDC5 cells inhibited LPS-induced inflammation and apoptosis, as evidenced by decreased production of TNF-α, IL-1β, IL-6, and ROS, together with a reduced ratio of TUNEL-positive cells, a lower expression of Bax and cleaved caspase 3, but a higher expression of Bcl-2. Meanwhile, SFRP5 overexpression also repress Wnt5a and phosphorylated JNK expression. However, the overexpression of Wnt5a considerably weakened the inhibitory effect of SFRP5 on LPS-triggered inflammation and apoptosis. Besides, the level of Wnt5a and JNK phosphorylation, which was inhibited by SFRP5 overexpression, was also partially recovered by Wnt5a overexpression.

**Conclusion:**

SFRP5 could alleviate LPS-induced ATDC5 cell inflammation and apoptosis; these actions may rely on repressing Wnt5a/JNK activation.

## Background

Osteoarthritis (OA) is a common chronic joint disease characterized by degeneration of articular cartilage and secondary bone hyperplasia [1]. The involved tissues of OA mainly include synovium, cartilage tissue, and subchondral bone, especially the pathological changes of cartilage, the specific manifestations of which are destruction of articular cartilage, osteophyte formation, synovitis, and narrowing of the joint space [2, 3]. The risk factors for OA include age, gender, occupation, obesity, diet, metabolic pathways, socioeconomic status, and family medical history, but its exact pathogenesis is not yet clear [4]. In recent years, it has been demonstrated that chondrocytes’ inflammation and apoptosis, degradation of extracellular matrix, and reconstruction of subchondral bone are the main causes of OA [5]. In particular, the apoptosis and inflammation of chondrocyte plays a key role in the progression of OA [6].

Secreted frizzled-related protein 5 (SFRP5) is a member of the SFRPs family, encoded by the SFRP gene and secreted by white adipose tissue [7]. The structure of SFRPs contains a netrin-like functional domain and a cysteine-rich domain, both of which are high homologous with the cysteine-rich domain of coiled protein and can compete with coiled protein to bind wingless-type family (Wnt) ligands, thus exerting a negative regulatory effect on the Wnt pathway [8]. SFRP5 has been identified as an anti-inflammatory adipokine, an endogenous inhibitor of Wnt5a signaling [8, 9]. Previous studies unanimously reported that SFRP5 exerted an anti-inflammatory effect by inhibiting the non-canonical Wnt5a/ c-Jun N-terminal kinase (JNK) signaling pathway, therefore playing a key role in repressing the occurrence and development of various diseases including obesity, cardiovascular diseases, and diabetes [10, 11]. For example, SFRP5 was found to diminish cardiac inflammation and protect the heart from ischemia/reperfusion injury via inactivating the non-canonical Wnt5a/JNK signaling [12]. Additionally, SFRP5 reduced the accumulation of activated macrophages in adipose tissue and suppressed the expression of pro-inflammatory cytokines such as TNF-α and IL-6 by inhibiting phosphorylation of JNK. However, little is known about the role of SERP5 in the development of OA. A previous study implicated that SFRP5 could suppress inflammatory response in rheumatoid arthritis fibroblast-like synoviocytes through downregulating JNK [13]. Intriguingly, the Wnt/JNK pathway has been extensively documented to be involved in the progression of OA [14, 15].

Given these above observations, it would be reasonable to assume that SFRP5 may have an anti-inflammatory role in LPS-stimulated chondrocytes, which is one of the pathological mechanisms of OA. We hypothesized that SFRP5 would affect the expression and production of pro-inflammatory mediators and apoptotic proteins in LPS-induced ATDC5 cells, via inactivating Wnt5a/JNK pathway.

## Methods

### Cell culture and treatment

Mouse chondrocytes ATDC5 cell line was obtained from Riken Cell Bank (Tsukuba, Japan). The cells were cultured in a 1:1 blend of Dulbecco’s modified Eagle’s medium and Ham’s F-12 medium (DMEM/F-12; GIBCO, USA) supplemented with 10% fetal bovine serum (GIBCO), in a wet incubator containing 5% CO_2_ and 95% air at 37 °C. Culture medium was refreshed every 3 days. Then, the cells were exposed to increasing concentrations of LPS (0, 1, 5, and 10 μg/ml) at 37 °C for 24 h.

### Cell transfection

The full-length PCR product of wild-type SFRP5 or Wnt5a was cloned into pcDNA3.1 vector (ov-SFRP5/ ov-Wnt5a) or the empty vector (ov-NC) with PFU DNA polymerase (Beyotime Biotechnology Co., LTD, China). For transient transfection experiments, ATDC5 were plated in a 24-well plate at a density of 2 × 10^5^ for 24 h prior to transfection. Lipofectamine 2000 (Thermo Fisher Scientific, USA) was used to perform transfection with 2.0 mg pcDNA3.1 SFRP5/Wnt5a vector or 2.0 mg pcDNA3.1 empty vector according to the manufacturer’s instructions.

### Cell counting kit-8 (CCK-8)

After treatment, cell viability was determined via CCK-8 assay (Beyotime). The cells were seeded in a 96-well plate at a density of 5 × 10^3^ cells/well and incubated overnight at 37 °C. Next, 10 μl of CCK-8 solution was added and the cells were cultured for 1.5 h at 37 °C. The absorbance at 450 nm was measured in a microplate reader (Bio-Rad, CA).

### Enzyme-linked immunosorbent assay (ELISA)

The concentration of TNFα, IL-1β, and IL-6 in the culture supernatants of ATDC5 cells was determined using commercially available ELISA kits (R&D Systems) according to the manufacturer’s instruction. The absorbance was read using microplate reader (SpectraMax 340; Molecular Devices Co., USA) at a wavelength of 450 nm.

### Measurement of reactive oxygen species (ROS)

The concentration of ROS in ATDC5 cells was determined by commercially available ROS kit (Abcam, UK) according to the manufacturer’s instruction. In brief, 2.5 × 10^4^ cells were seeded on a 96-well plate. After allowing to grow overnight, cells were washed with 1× Buffer and then stained with 25 μM DCFDA for 45 min at 37 °C. The signal was read at the absorbance of 485/535 nm.

### Terminal deoxynucleotidyl transferase-mediated dUTP nick-end labeling (TUNEL) staining

The apoptotic ATDC5 cells were stained using TUNEL assay kit (KeyGen, Biotechnology, Co., LTD, China) according to the manufacturer’s protocol. Briefly, cells seeded on the slides were fixed with 4% paraformaldehyde at room temperature for 0.5 h. After treatment with 1% Triton X-100/10 mM PBS for 5 min, proteinase K was added. Then, cells were incubated with TdT enzyme reaction solution, streptavidin-HRP working solution, and DAB staining working solution in the dark, respectively. Hematoxylin was used to stain the nucleus.

### Western blot analysis

ATDC5 cells were homogenized in RIPA lysis buffer containing protease inhibitors (Roche, China) for 30 min, then centrifuged at 4 °C to separate the lysates. The concentration and purity were determined by BCA kit (Solarbio, China). Next, 30 mg of protein was transferred onto PVDF membranes (Millipore, USA) and blocked with non-fat milk at room temperature for 2 h. The membrane was then incubated with primary antibodies against SFRP5, Bcl-2, Bax, Caspase 3, Wnt5a, JNK, phosphorylated (p)-JNK, and GAPDH (Santa Cruz Biotechnology, USA). The secondary antibodies were conjugated with horseradish peroxidase (Santa Cruz) and incubated for immunoblotting analysis. Signal detection was measured using an ECL Western blotting detection kit (Amersham Biosciences, USA). The expression of the target gene was represented relative to GAPDH.

### Real-time quantitative PCR (RT-qPCR)

Total RNA from ATDC5 cells was extracted using TRIzol reagent (Invitrogen, USA), and concentrations were measured by a NanoDrop (R&D Systems, USA). Total RNA was reversely transcribed into cDNA using PrimeScript™ RT reagent Kit (TaKaRa, Biotechnology Co., LTD, China). QPCR was performed using 1 ml of complementary DNA (cDNA) per well, TaqMan Master Mix (Applied Biosystems, USA), and 250 nM each of sense and antisense primers. The primer used for qPCR to detect SFRP5 was as follows: forward, 5′-CTCCAGTGACTTTGTGGTCAAG-3′, reverse, 5′-TGTCTAA-CTGTGGGCAAGGG-3′; GAPDH: forward, 5′-CAGGAGAGTGTTTCCTCGTCC-3′, reverse, 5′-TTTGCCGTGAGTGGAGTCAT-3′. Results were evaluated using the CT method, and the calculated number of copies was normalized to the number of GAPDH mRNA copies in the same sample.

### Statistical analysis

All experiments were repeated at least three times. The results of multiple experiments were presented as mean ± standard deviation (SD). Statistical analysis was conducted using GraphPad 6.0 statistical software (GraphPad, USA). Differences among groups were assessed using one-way analysis of variance (ANOVA). *P* < 0.05 was considered statistically significant.

## Results

### SFRP5 is downregulated upon LPS stimulation in ATDC5 cells

**Fig. 1:**
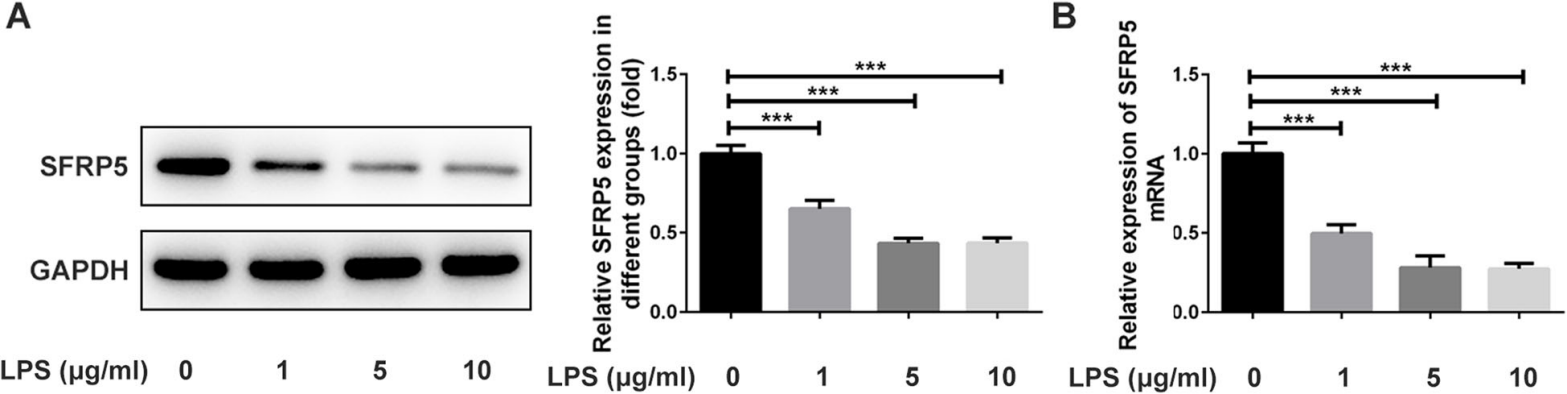
SFRP5 is downregulated upon LPS stimulation in ATDC5 cells. The protein (**a**) and mRNA (**b**) level of SFRP5 in ATDC5 cells that exposed to different concentrations of LPS. ***P < 0.001

First of all, to determine whether SFRP5 could participate in LPS-triggered damage in ATDC5 cells, we compared the expression of SFRP5 in ATDC5 before and after increasing concentrations of LPS treatment. As shown in Fig. 1a, compared to control cells, cells that subjected to LPS with the concentrations of 1, 5, and 10 μg/ml exerted significantly lower protein expression of SFRP5. The same result was observed in Fig. 1b, which presented the alterations of SFRP5 mRNA level upon LPS stimulation. These results implicated that, consistent with our speculation, SFRP5 may play a role in LPS-induced inflammation and apoptosis in ATDC5 cells. In addition, LPS also resulted in a decrease in cell viability, and LPS with the concentration of 5 μg/ml was chosen for subsequent experiment, considering that the effect of LPS on SFRP5 expression and cell viability reached the highest at the concentration of 5 μg/ml (Fig. 1c).

### Overexpression of SFRP5 inhibits LPS-triggered inflammation and apoptosis in ATDC5 cells

**Fig. 2:**
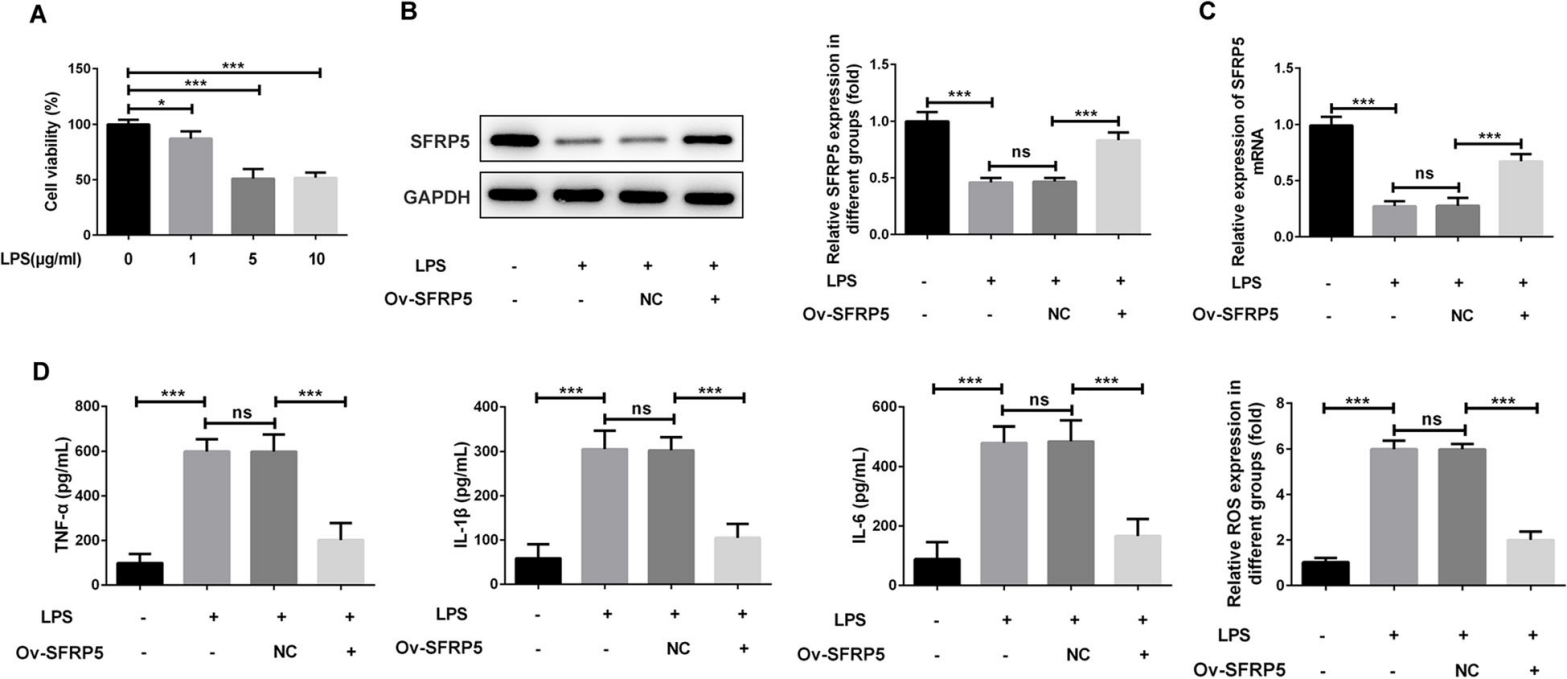
Overexpression of SFRP5 inhibits LPS-triggered inflammation in ATDC5 cells. **a** The cell viability of ATDC5 cells that exposed to different concentrations of LPS. **b**, **c** The protein (**b**) and mRNA (**c**) level of SFRP5 in ATDC5 cells before and after SFRP5 overexpression in the presence of 5 μg/ml LPS treatment. **d** The concentration of inflammatory cytokines including TNF-α, IL-1β, and IL-6 in the culture supernatants of ATDC5 cells. **e** The level of ROS relative to control group in ATDC5 cells. *P < 0.05, ***P < 0.001. Ov, overexpression; NC, negative control; ns, no significance

Next, we overexpressed SFRP5 in ATDC cells, and Ov-NC was used as negative control. Results from Fig. 2a and b verified the successful overexpression of SFRP5 in ATDC5 cells. Subsequently, the generation of inflammatory cytokines including TNF-α, IL-1β, and IL-6 together with ROS in ATDC5 cells that overexpressed with SFRP5 or not was measured. As shown in Fig. 2c and d, LPS resulted in an obvious increase in TNF-α, IL-1β, IL-6, and ROS. However, overexpression of SFRP5 remarkably reduced the concentrations of these cytokines.

**Fig. 3:**
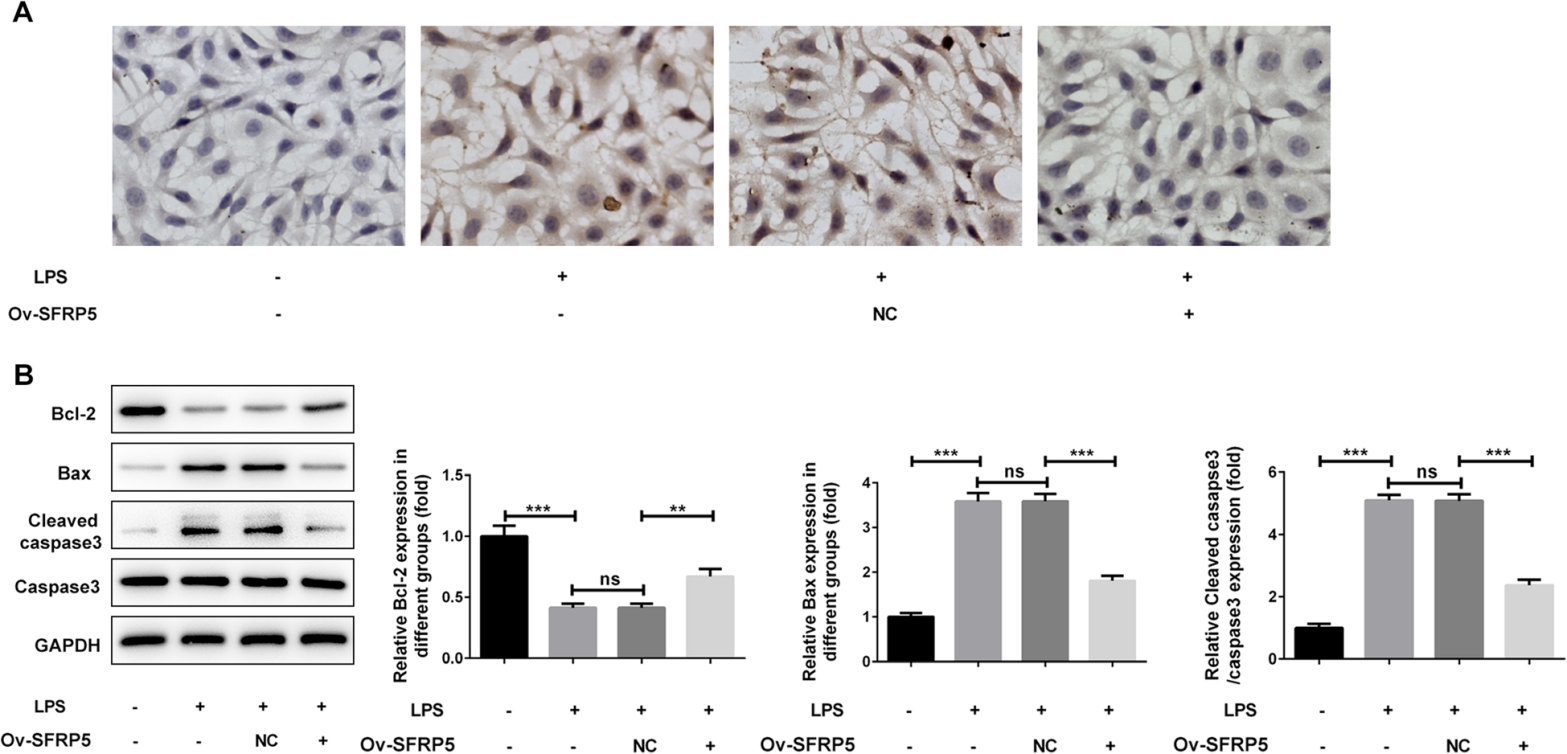
Overexpression of SFRP5 inhibits LPS-triggered apoptosis in ATDC5 cells. **a** Representative images for TUNEL staining, apoptotic cells were stained with dark brown (× 200). **b** Representative blots and quantitative analysis for Bcl-2, Bax, and cleaved caspase 3. **P < 0.01, ***P < 0.001. Ov, overexpression; NC, negative control; ns, no significance

TUNEL staining was utilized to observe apoptotic cells. As shown in Fig. 3a, LPS treatment caused a higher ratio of apoptotic cells, which was instead reversed by SFRP5 overexpression, indicating the inhibitory effect of SFRP5 overexpression on LPS-induced apoptosis. The same result is observed in Fig. 3b, LPS stimulation enhanced pro-apoptotic proteins Bax and cleaved caspase 3 expression, but reduced anti-apoptotic protein Bcl-2 expression. Nevertheless, SFRP5 overexpression partially recovered the expression of Bcl-2, Bax, and cleaved caspase 3.

### Overexpression of SFRP5 represses Wnt5a expression and phosphorylation of JNK

**Fig. 4:**
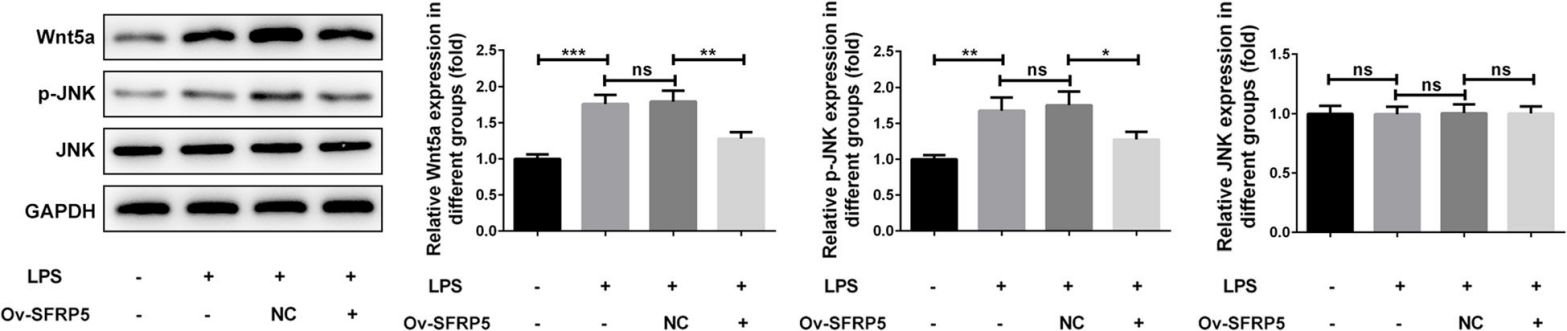
Overexpression of SFRP5 represses Wnt5a expression and phosphorylation of JNK. The representative blots and quantitative analysis for Wnt5a, JNK, and phosphorylated JNK. **P < 0.01, ***P < 0.001. Ov, overexpression; NC, negative control; ns, no significance

Then, we measured the expression of Wnt5a and (p)-JNK in response to SFRP5 overexpression. As demonstrated in Fig. 4, LPS stimulation considerably activated Wnt5a and p-JNK expression; however, overexpression of SFRP5 significantly inhibited Wnt5a and p-JNK expression, compared to LPS treatment. These data indicated that Wnt5a may play a role in the actions of SFRP5 on LPS-stimulated ATDC5 cells.

### Overexpression of Wnt5a blunts the inhibitory effect of SFRP5 elevation on LPS-induced inflammation and apoptosis in ATDC5 cells

**Fig. 5:**
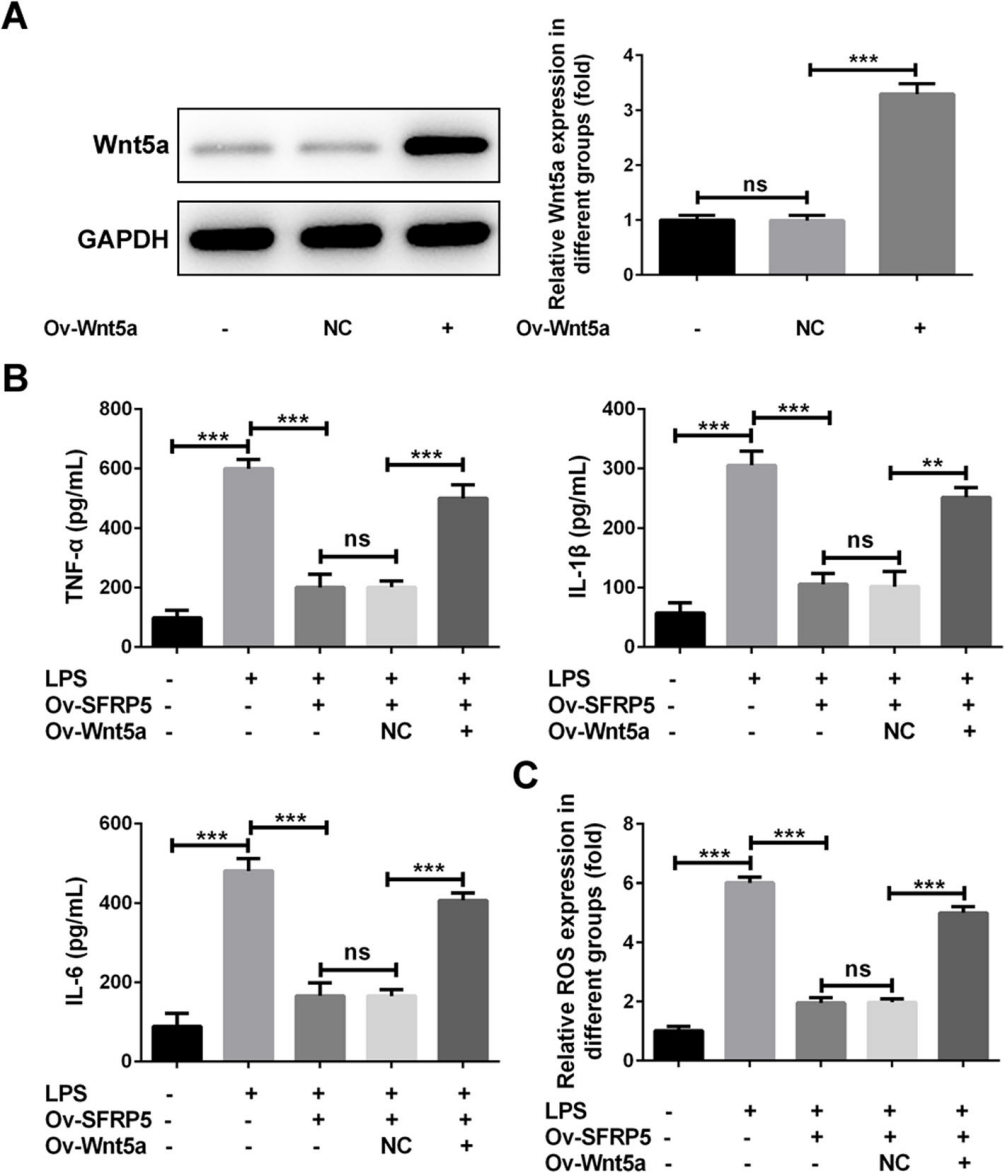
Overexpression of Wnt5a blunts the inhibitory effect of SFRP5 elevation on LPS-induced inflammation in ATDC5 cells. **a** The protein expression of Wnt5a in ATDC5 cells or cells that transfected with NC or ov-Wnt5a vectors. **b** The concentration of inflammatory cytokines including TNF-α, IL-1β, and IL-6 in the culture supernatants of ATDC5 cells. **c** The level of ROS relative to control group in ATDC5 cells. **P < 0.01, ***P < 0.001. Ov, overexpression; NC, negative control; ns, no significance

**Fig. 6:**
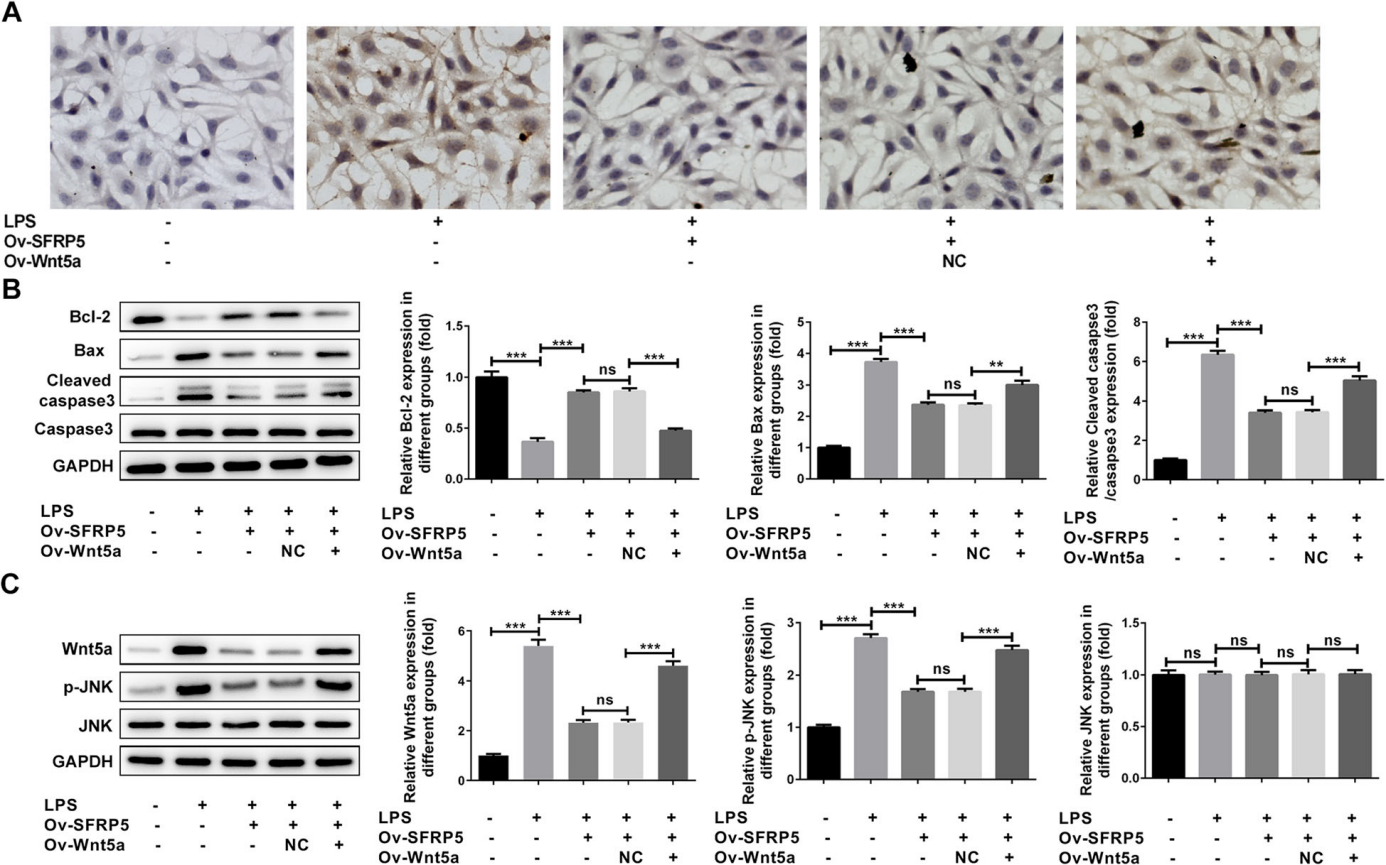
Overexpression of Wnt5a blunts the inhibitory effect of SFRP5 elevation on LPS-induced apoptosis in ATDC5 cells. **a** Representative images for TUNEL staining, apoptotic cells were stained with dark brown (× 200). **b** Representative blots and quantitative analysis for Bcl-2, Bax, and cleaved caspase 3. **c** The representative blots and quantitative analysis for Wnt5a, JNK, and phosphorylated JNK. **P < 0.01, ***P < 0.001. Ov, overexpression; NC, negative control; ns, no significance

Finally, to confirm whether SFRP5 blocked LPS-triggered inflammation and apoptosis through inactivating Wnt5a/JNK pathway. We overexpressed Wnt5a and SFRP5 into LPS-challenged ATDC5 cells simultaneously (Fig. 5a). In accordance with the above observations, SFRP5 overexpression significantly prevented LPS-induced inflammation and apoptosis (Figs. 5 and 6). However, compared to cells that transfected with Ov-NC, cells that overexpressed with Wnt5a produced elevated concentrations of TNF-α, IL-1β, IL-6, and ROS (Fig. 5b and c). The ratio of apoptotic cells together with the expression of Bcl-2, Bax, and cleaved caspase 3 were also reversed to close to LPS group, in comparison to LPS + SFRP5 overexpression (Fig. 6a and b). Furthermore, Wnt5a overexpression enhanced the expression of Wnt5a and p-JNK, as compared to LPS + SFRP5 overexpression (Fig. 6c).

## Discussion

The evidence accumulated to date demonstrates that the Wnt signaling pathway plays a crucial role in the pathogenesis of OA. Wnt family proteins are a class of morphogens involved in embryonic skeletal formation, tissue repair, fibrosis, and joint homeostasis [16]. Wnt regulates multiple signaling cascades, including the canonical Wnt-β catenin pathway, which is usually quiescent in many adult organs but can be activated in response to injury [17, 18]. In the pathogenesis of OA, activation of canonical Wnt signaling is observed in both articular cartilage and synovium following injury, with upregulated expression of both Wnt ligands and target genes [19, 20]. It has been shown that overexpression of β-catenin induced in mature chondrocytes can exacerbate cartilage degeneration, chondrocyte hypertrophy, and matrix proteinase expression [21]. Inhibition of Wnt/β-catenin signaling could ameliorate OA in animal’s model of experimental OA [22]. Expressions of Wnt5a in articular cartilage has been positively correlated to progressive damage of knee OA joints [23]. An in vitro study found that Wnt5a promoted inflammatory response via activating β-catenin independent signaling including JNK in human OA cartilage [24]. Taken together, these findings suggest that the Wnt5a-mediated Wnt signaling pathway participates in promoting pro-inflammatory cytokine and chemokine production in the development of OA.

In the present study, we found the presence of SFRP5 mRNA and protein in ATDC5 chondrocytes. Consistently, Kwon et al. showed that SFRP5 mRNA expressions were also detected in fibroblast-like synoviocytes from patients with OA [13]. The data from the present study and others provide evidence for SFRP5 expression in articular synovium as well as the potential role of SFRP5 in OA.

In the current study, we also demonstrated that the level of both SFRP5 mRNA and protein was markedly downregulated upon LPS stimulation and that the expression of various pro-inflammatory cytokines such as TNF-α, IL-1β, IL-6, and ROS together with the cell apoptosis triggered by LPS injury was considerably suppressed by overexpression of SFRP5 in ATDC chondrocytes. Previous data suggested that decreased SFRP5 gene level promoted the production of matrix metalloproteinase (MMP) and lead to the migration and metastasis of gastric cancer cells [25]. Circulating SFRP5 expression was also lower in patients with impaired glucose tolerance or type 2 diabetes mellitus and inversely correlated with markers for obesity [11, 26]. Like these previous observations, our data indicated that as a negative regulator of Wnt signaling, SFRP5 is downregulated in LPS-treated chondrocytes and has anti-inflammatory effects. Our data provide evidence for the anti-inflammatory effect of SFRP5 in OA.

SFRP5 can antagonize Wnt5a signaling, which contributes to the production of pro-inflammatory cytokines and apoptosis in chondrocytes [27]. On the other hand, it has been shown that enhanced Wnt signaling inhibits the level of SFRP5 mRNA. This negative feedback mechanism leads to a further increase in Wnt signaling as SFRP5 expression decreases [28]. In this study, the anti-inflammatory and anti-apoptotic effect of SFRP5 was remarkably blunted by the overexpression of Wnt5a, suggesting that SFRP5 alleviated LPS-induced chondrocytes injury via inhibiting Wnt5a expression. Furthermore, we found that the phosphorylated form JNK, a downstream target of non-canonical Wnt signaling, was downregulated by SFRP5 overexpression in ATDC5 cells. These results demonstrated that overexpression of SFRP5 in chondrocytes reversed the activated Wnt/JNK signaling and inhibited the expression of pro-inflammatory mediators as well as occurrence of apoptosis induced by LPS, indicating that the SFRP5/Wnt5a/JNK regulatory axis represents a potential target for controlling LPS-induced injury in chondrocytes and even the progression of OA.

## Conclusions

In conclusion, the present study showed significant effects of SFRP5 regulation on the inflammatory response and apoptosis in chondrocytes as well as the downregulation of JNK signaling. Although the exact mechanisms underlying remain to be further clarified, our findings indicate a novel role for SFRP5 as a negative regulator in chondrocytes injury and support its potential role in the treatment of OA.

## Data Availability

All data generated or analyzed during this study are included in this published article.

## Abbreviations

OA: Osteoarthritis
SFRP5: Secreted frizzled-related protein 5
Wnt: Wingless-type family
JNK: c-Jun N-terminal kinase
CCK-8: Cell counting kit-8
ELISA: Enzyme-linked immunosorbent assay
ROS: Reactive oxygen species
TUNEL: Terminal deoxynucleotidyl transferase-mediated dUTP nick-end labeling
